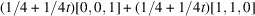# Dynamic correlations and possible diffusion pathway in the superionic conductor Cu_2−*x*_Se

**DOI:** 10.1107/S2052252523001318

**Published:** 2023-02-17

**Authors:** Nikolaj Roth, Bo Brummerstedt Iversen

**Affiliations:** a Center for Integrated Materials Research, Department of Chemistry and iNano, Aarhus University, Aarhus 8000, Denmark; UCL, United Kingdom

**Keywords:** superionic conductors, three-dimensional difference pair distribution function analysis, phonon-liquid electron-crystals, diffusion pathways

## Abstract

The superionic conductor Cu_2−*x*_Se has regained interest as a thermoelectric material owing to its low thermal conductivity, suggested to arise from a liquid-like Cu substructure, and the material has been coined a phonon-liquid electron-crystal. Using high-quality three-dimensional X-ray scattering data measured up to large scattering vectors, accurate analysis of both the average crystal structure as well as the local correlations is carried out to shed light on the Cu movements. The Cu ions show large vibrations with extreme anharmonicity and mainly move within a tetrahedron-shaped volume in the structure. From the analysis of weak features in the observed electron density, the possible diffusion pathway of Cu is identified, and it is clear from its low density that jumps between sites are infrequent compared with the time the Cu ions spend vibrating around each site. These findings support the conclusions drawn from recent quasi-elastic neutron scattering data, casting doubt on the phonon-liquid picture. Although there is diffusion of Cu ions in the structure, making it a superionic conductor, the jumps are infrequent and probably not the origin of the low thermal conductivity. From three-dimensional difference pair distribution function analysis of the diffuse scattering data, strongly correlated movements are identified, showing atomic motions which conserve interatomic distances at the cost of large changes in angles.

## Abstract 2

Very high-resolution X-ray scattering data are used to study the disordered structure of Cu_2−*x*_Se in the high-temperature superionic phase.

## 1. Introduction

A superionic conductor (also called a fast-ion conductor or solid electrolyte) is a solid that displays ionic conductivities that are typical of those found in molten salts, *i.e.* on the order of 1 Ω^−1^ cm^−1^ (Boyce & Huberman, 1979 ▸). This is in contrast to most normal ionic solids which have much lower ionic conductivities and, as an example, NaCl has an ionic conductivity of less than 10^−8^ Ω^−1^ cm^−1^ at 200 K (Boyce & Huberman, 1979 ▸). Superionic conductors typically have a relatively ordered structure of one or several types of ions with enough space to allow mobile ions of a disordered substructure to pass through. The usual description of a structure as a collection of discrete positions then breaks down. The space occupied by the mobile ions in the periodic average crystal structure will form continuous channels, although these might be very difficult to observe or measure depending on the frequency of movements through the channels.

Depending on the Cu content of Cu_2−*x*_Se there is a phase-transition from the low-temperature β-Cu_2−*x*_Se to α-Cu_2−*x*_Se in the range 350–414 K (Brown *et al.*, 2013 ▸, 2016 ▸; Eikeland *et al.*, 2017 ▸; Vucic *et al.*, 1984 ▸; Liu, Shi*, et al.*, 2013 ▸; Liu, Yuan*, et al.*, 2013 ▸). The high-temperature α-phase is a superionic conductor (Miyatani, 1973 ▸; Vucic *et al.*, 1982 ▸; Mahan, 2015 ▸; Sirusi *et al.*, 2015 ▸; Kang *et al.*, 2016 ▸). In stoichiometric Cu_2_Se the transition temperature is 414 K and the ionic conductivity is about 2 Ω^−1^ cm^−1^ at 600 K. The transition temperature decreases gradually with composition parameter *x*, and the transition vanishing at *x* ≃ 0.25 (Yakshibaev & Balapanov, 1984 ▸). α-Cu_2−*x*_Se has a total thermal conductivity of around 0.9 W m^−1^ K^−1^ (Zhao *et al.*, 2020 ▸) with a lattice contribution of around 0.6 W m^−1^ K^−1^ (Liu *et al.*, 2012 ▸), which is close to the thermal conductivity of water at room temperature (0.6 W m^−1^ K^−1^) or ordinary soda-lime glass (in the range 0.8 to 1 W m^−1^ K^−1^). It was proposed that the low thermal conductivity is a result of a liquid-like Cu substructure, and the material was coined a phonon-liquid electron-crystal (Liu *et al.*, 2012 ▸). Liquids have low thermal conductivities as a consequence of them not propagating shear waves, and the phonon-liquid concept relies on the loss of transverse acoustic and optical modes. If the Cu substructure is liquid-like, clear channels of electron density should be observable in the crystal structure.

The structure of α-Cu_2−*x*_Se is generally described as a variation of the anti-fluorite structure with a face-centered cubic (f.c.c.) arrangement of Se and Cu in all the tetrahedral sites. Although all studies agree that Se is ordered and located on the f.c.c. 4*a* sites, the Cu substructure has some degree of disorder. Many studies use a split-site model for Cu, where the Cu is distributed on the tetrahedral 8*c* sites as well as on 32*f* sites, which form the corners of a tetrahedron around the 8*c* sites, with corners pointing into the octahedral 4*b* sites. The 4*a*, 4*b* and 8*c* sites are fixed [4*a*(0,0,0); 4*b*(1/2,1/2,1/2); 8*c*(1/4,1/4,1/4)], however the 32*f* site (*x*,*x*,*x*) has a degree of freedom. Different papers have reported different values of *x*, 1/4 < *x* < 1/2, *i.e.* how far reaching the corners of the tetrahedron around the 8*c* site are. This seems to correlate with the refined occupancies of the sites together with the thermal parameters (Danilkin *et al.*, 2012 ▸, 2011 ▸; Yamamoto & Kashida, 1991 ▸; Oliveria, 1988 ▸; Eikeland *et al.*, 2017 ▸). An example of this typical split-site model for Cu can be seen in Fig. 2(*a*).

Though some studies interpret the discrete Cu positions in the split-site model as a physical reality, the general agreement in crystallographic studies is that the split-site model is used to model a continuous density of Cu. Yamamoto & Kashida (1991 ▸) employed single-crystal X-ray diffraction to determine the average structure (Yamamoto & Kashida, 1991 ▸). They measured a total of 26 unique reflections for α-Cu_2−*x*_Se and refined the structure using a split-site model with subsequent analysis of the density obtained by Fourier inversion. They found 95% of the Cu-density to be distributed on the 8*c* and 32*f* sites consisting of a large maximum on the 8*c* position with long tails along the 〈111〉 directions through the 32*f* sites. The remaining 5% of Cu was modeled occupying the octahedral 4*b* site. This density on the octahedral 4*b* site has not been reproduced in other high-quality single-crystal studies. However, the agreement between equivalent reflections was low, giving a high *R*_int_ of 9.7%, suggesting that low data quality might be the origin of the 5% Cu density at the octahedral site. Furthermore, the 5% Cu on the octahedral site refined to have a thermal parameter four times as large as the 8*c* site, showing this to describe a very broad and spread-out density.

The highest-quality dataset so far was reported by Oliveria (1988 ▸) using single-crystal neutron diffraction, where 34 unique reflections were measured with an agreement factor between equivalent reflections of *R*_int_ = 4.0%. The dataset was modeled using a split-site model with anharmonic Gram–Charlier coefficients to the sixth order. The density obtained from Fourier inversion shows the Cu density as a large maximum on the tetrahedral 8*c* position with long tails along the 〈111〉 directions through the 32*f* sites, but no significant density on the octahedral site. The same was found in a recent study by Dalgaard *et al.* (2018 ▸), where single-crystal X-ray diffraction data (22 unique reflections, *R*_int_ = 7.9%) were analyzed using the maximum entropy method. The structure of the low-temperature β-Cu_2−*x*_Se was recently shown to be ordered in two dimensions and disordered in one, consisting of ordered layers with a disordered stacking sequence (Roth & Iversen, 2019 ▸). The relation between the average structure in the α and the β phase was reported by Eikeland *et al.* (2017 ▸).

So far, the studies on the average structure of α-Cu_2−*x*_Se have shown Cu to be distributed in tetrahedron-shaped volumes with corners pointing toward the empty octahedral sites, but no direct sign of the Cu diffusion pathway has been found in the average structure. The dataset presented here contains 115 unique reflections with *R*_int_ = 3.06%, allowing for a more detailed analysis of the average structure.

Several studies have examined the deviations from the average structure in α-Cu_2−*x*_Se. Cava *et al.* (1986 ▸) measured neutron diffuse scattering on single crystals of α-Cu_2−*x*_Se in the *hhl* plane. Although very limited analysis of the dataset was carried out, they found differences in the diffuse scattering that were stoichiometry-dependent. To further investigate the origin of the diffuse scattering, they measured quasi-elastic neutron scattering data, *i.e.* measuring with a high enough energy-resolution to resolve elastic processes from low-energy modes such as diffusion processes. They found the diffuse scattering to mainly be of dynamic origin.

Danilkin *et al.* (2012 ▸, 2011 ▸) measured quasi-elastic neutron scattering and neutron diffraction on powder samples of several compositions of α-Cu_2−*x*_Se. They found the diffuse scattering to be mainly quasi-elastic, again showing the dynamic origin. By analysis of the *q*-dependence of the diffuse scattering, they proposed that both nearest Se–Cu and Cu–Cu correlations contribute strongly to the signal. From the broadening of the quasi-elastic spectra they were able to estimate the residence time for Cu between jumps and the jump length. Depending on stoichiometry the residence time was estimated to between 1 and 4 ps (with shorter residence times for stoichiometry closer to Cu_2_Se) and jump length between 3 and 4 Å. This jump length would be consistent with jumps between nearest Cu sites. They further proposed that the diffusion path of Cu goes from the tetrahedral site along the 〈111〉 directions toward the octahedral 4*b* site, but then avoids going through the 4*b* site by changing direction to 〈100〉 in its vicinity, followed by 〈111〉 to one of the nearest tetrahedral sites. Danilkin *et al.* (2009 ▸) also published diffuse neutron scattering measurements on a single crystal of α-Cu_1.8_Se in the *hhl* plane. By measuring energy-dispersive data they once again showed the diffuse scattering to have a largely dynamic origin, although without any further analysis of the scattering.

Voneshen *et al.* (2017 ▸) also studied Cu diffusion through quasi-elastic neutron scattering. In contrast to the studies by Danilkin *et al.* (2009 ▸) they model the data using several residence times for different jumps. For jumps within a single Cu tetrahedron they estimate a residence time of 0.3–2 ps in the α-phase. They argue that these very frequent and short jumps, which do not contribute to diffusion, are a sign of anharmonicity and not jumps between discrete sites. For jumps between nearest Cu sites, which allow Cu diffusion, they find a residence time between 10 and 20 ps, depending on temperature.

With such long residence times for jumps between tetrahedral sites, Voneshen *et al.* (2017 ▸) argue that the phonon-liquid concept is invalid, as the hopping time scales are too slow to significantly affect lattice vibrations. They further measured the projected phonon density of states and showed that the transverse phonons persist at all temperatures, which is inconsistent with the phonon-liquid picture. They argue that the low thermal conductivity instead arises from the extreme anharmonicity in α-Cu_2−*x*_Se. These conclusions have also been underlined by Kumar *et al.* (2022 ▸) using inelastic and quasi-elastic neutron scattering as well as *ab initio* molecular dynamics simulations. They find that phonon modes involving Cu vibrations are well defined up to very high temperatures, that the Cu diffusion is solid-like and attribute the low thermal conductivity to anharmonicity. Another argument against the phonon-liquid picture is the thermal conductivity, which does not change significantly between the low-temperature non-ion-conducting β-Cu_2−*x*_Se and the high-temperature superionic conductor α-Cu_2−*x*_Se [see for example the review by Zhao *et al.* (2020 ▸) where the thermal conductivities across the phase transition from different studies are shown]. The lattice contribution to the thermal conductivity was found to be around 0.6 W m^−1^ K^−1^ [see the supporting information of Liu *et al.* (2012 ▸)]. This is higher than for the clathrate Ba_8_Ga_16_Ge_30_ at the same temperature [around 0.3 W m^−1^ K^−1^ at 450 K (Toberer *et al.*, 2008 ▸)]. Ba_8_Ga_16_Ge_30_ is generally considered a phonon-glass electron-crystal material which has a low thermal conductivity from rattler modes (Christensen *et al.*, 2008 ▸).

Thus, although Cu diffusion in α-Cu_2−*x*_Se makes it a superionic conductor, some results from the existing literature suggest that the thermoelectric properties might be better described using the phonon-glass electron-crystal concept rather than the recently coined phonon-liquid electron-crystal term. Similar findings have been made for other systems originally coined as phonen-liquids, casting doubt on the general validity of the concept (Rettie *et al.*, 2018 ▸; Niedziela *et al.*, 2019 ▸; Ding *et al.*, 2020 ▸).

**Figure 1:**
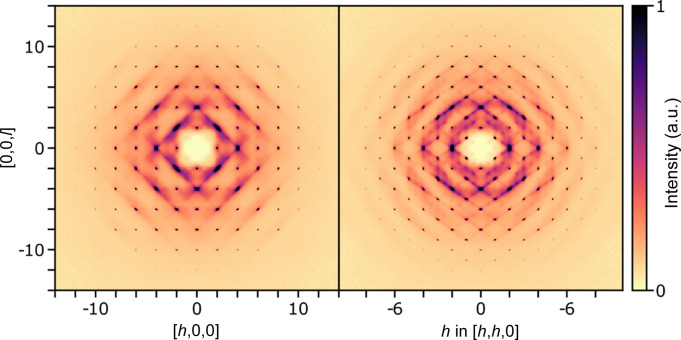
Total scattering in α-Cu_2−*x*_Se at 401 K in the *hk*0 plane (left) and *hhl* plane (right). Data are shown for a limited range for clarity but the measured data extends to ±30 Å^−1^.

In the following sections, the correlated movements and the diffusion pathway in α-Cu_2−*x*_Se will be examined. To do this, three-dimensional reciprocal space X-ray scattering data has been measured up to large scattering vectors (*q* = 30 Å^−1^). The measured total scattering for α-Cu_2−*x*_Se at 401 K is shown in Fig. 1 ▸ for the *hk*0 and *hhl* planes with a limited *q*-range for clarity. This high *q*-range allows analysis of fine details of both the average structure as well as the correlations. In addition to the high *q*-range, a large dynamic range of scattering intensity has been measured through several configurations of beam attenuation and exposure time. This further allows determination of very weak scattering, both the diffuse scattering but also the Bragg intensities, which decay quickly with *q* as a consequence of the large atomic movements.

## 2. Experimental

Single crystals of Cu_2−*x*_Se were grown using chemical vapor transport (CVT) with iodine as a transport agent as previously reported by Eikeland *et al.* (2017 ▸). A single crystal of Cu_2−*x*_Se (around 60 µm octahedron) was glued to the end of a thin glass pin using ep­oxy and mounted on a goniometer. Data were measured at the BL02B1 beamline at the SPring-8 synchrotron using a photon energy of 50.00 keV and a sample-to-detector distance of 130 mm on a Huber four-circle (quarter χ) goniometer equipped with a Pilatus3 X 1M CdTe (P3) detector. The crystal was mounted at room temperature in the β-phase and heated to 401 K using a nitro­gen streamer calibrated using a thermocouple in the crystal position. At this temperature the phase transition to α-Cu_2−*x*_Se occurred.

First a dataset for the strongest reflections was collected. The used Dectris Pilatus 1M CdTe detector is essentially a single-photon counting detector. However, if the photon flux becomes high the detector will miss some photons due to multiple photons hitting at the same time. The detector software can correct for this to some degree, unless the flux becomes too high as discussed by Krause *et al.* (2020 ▸). To avoid this issue, a few test measurements were carried out to estimate the flux of the most intense peak. The beam was then attenuated using a 250 µm Ta film (giving 12% transmission at 50 keV) to avoid a too-high photon flux. Note that this effect is different from the saturation of the detector. Data were measured during a continuous 180° Ω rotation with χ = 0° and the detector at 2θ = 0°. In total, 900 frames were measured (each frame spans 0.2°) with an exposure time of 0.4 s per frame. Second, the intermediate range intensities were collected without the beam attenuator. Here four runs were measured, each a 180° Ω rotation with 900 frames, for χ = 0° and χ = 45°, with the detector at 2θ = 0° and 2θ = 30°. An exposure time of 0.4 s per frame was used. Third, the very weak scattering was measured without any beam attenuation. Here four runs were measured, each a 180° Ω rotation with 900 frames, for χ = 0° and χ = 45°, with the detector at 2θ = 0° and 2θ = 30°. An exposure time of 2 s per frame was used. Finally the background and air-scattering was measured using the same set of exposure times, detector positions and beam attenuation as those used for the crystal. For each combination of these, 200 frames of air scattering were measured.

To obtain the average structure the images were converted to the Bruker .sfrm format (Krause *et al.*, 2020 ▸) and integrated using *SAINT* (Bruker, 2013 ▸). Peaks with too-high estimated flux were cut from the frames without beam attenuation. The integrated data were processed and corrected using *SADABS* (Krause *et al.*, 2015 ▸) and subsequently merged using *SORTAV* (Blessing, 1997 ▸). Structural refinement was carried out using *JANA2006* (Petricek *et al.*, 2014 ▸). Anomalous scattering factors for Cu and Se at 50 keV were used in the refinement.

For the diffuse scattering analysis the dataset was converted to reciprocal space using a customized Matlab script, written by Martin von Zimmermann. During this process the dataset was corrected for Lorentz and polarization factors, the background scattering from air was subtracted, and a solid angle correction was applied as the detector is flat. The resulting data were symmetrized using the *m* 3 *m* point symmetry of the Laue group. The resulting scattering dataset was reconstructed on a 501 × 501 × 501 point grid with each axis spanning ±30.0 Å^−1^. For the production of a 3D-ΔPDF, the Bragg peaks were punched and filled. Because a large degree of diffuse scattering is observed at the positions of the Bragg peaks, care has to be taken to remove all Bragg scattering while leaving as much of the diffuse scattering as possible. To accomplish this, the Bragg peaks were removed directly from the raw data frames before conversion using a Python script which removes sharp and strong peaks from the data. This is possible as the diffuse scattering here varies slowly in space compared with Bragg peaks, which might not always be the case. The script compares the measured pixels to all neighboring pixels, and if a pixel is much stronger (a factor of 2.5 was used here) relative to its neighbors, it is marked as part of a Bragg peak. To avoid marking weak noise as peaks, a lower bound for the absolute intensity needed for a peak is also used. A high-intensity cutoff is also used, for which pixels larger than this value are automatically marked as peaks. Once peak pixels have been marked this way, the pixels neighboring these are also marked as being part of peaks. The peak pixels found were then filled using a two-dimensional spline interpolation to approximate the diffuse scattering at the Bragg position. This approach effectively removes most Bragg peaks, but leaves some of the almost-zero-intensity Bragg peaks at large scattering vectors. To also remove these, a small punch was applied to the allowed Bragg positions after conversion to reciprocal space and linear interpolation. The resulting dataset containing only the diffuse scattering was then Fourier transformed to give the three-dimensional difference pair distribution function (3D-ΔPDF). A further discussion on different punch types and sizes and their effects on the results is given in the supporting information.

## 3. Average crystal structure

The measured Bragg peaks contain a total of 11 303 reflections with *I* > 3σ. Of these there are 115 unique reflections, with an average redundancy of 98. This provides a complete Bragg dataset with a resolution of *d* = 0.403 Å (*q* = 15.7 Å^−1^). Although data were measured to higher *q*, the Bragg peaks beyond this are not observable due to a large Debye–Waller factor from the thermal motion. The equivalent reflections are in very good agreement, giving *R*_int_ = 3.06%, which is lower than in any previous studies. The reflection intensities, standard deviations and number of times each reflection was measured are given in the supporting information. A total of 115 unique reflections is considerably more than in any previous studies with the highest being 34 in the work by Oliveria (1988 ▸). The more than threefold increase in number of unique reflections together with the high data quality enables a more in depth analysis than previously possible.

**Figure 2:**
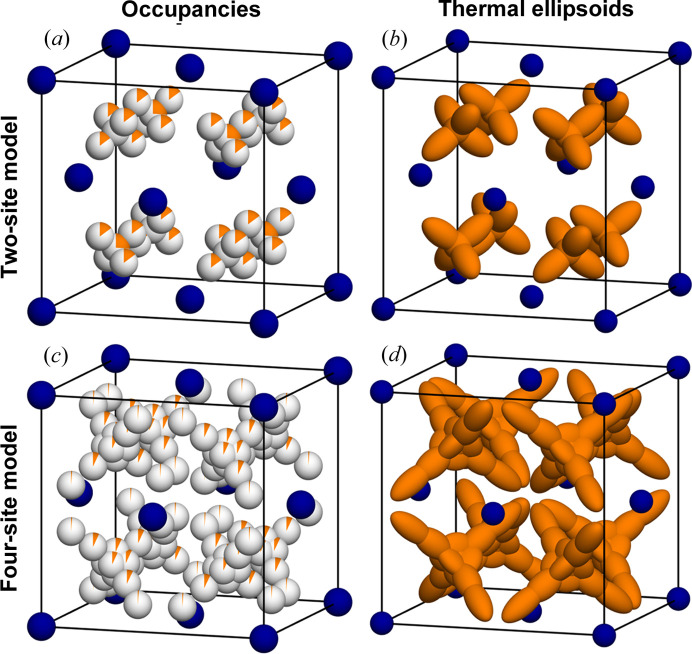
Average crystal structure models for α-Cu_2−*x*_Se at 401 K. (*a*) Two-site model with occupancies shown as partially colored spheres. (*b*) Two-site model with thermal ellipsoids. (*c*) Four-site model with occupancies shown as partially colored spheres. (*d*) Four-site model with thermal ellipsoids. Se are colored blue and Cu are colored orange

Three models for the average structure are refined against the measured data. First, the simple two-site model for Cu is refined. Here Cu is distributed on the central tetrahedral (8*c*) and trigonal (32*f*) sites. The Cu occupancies are refined freely and anisotropic thermal parameters are used. In total the model has seven parameters. Figs. 2 ▸(*a*) and 2 ▸(*b*) show the resulting structure and all parameters are listed in the supporting information. In Fig. 2 ▸(*a*) the atoms are shown as spheres with the partial coloring indicating the occupancies. In this model the central tetrahedral 8*c* site has an occupancy of 26% while that of the 32*f* sites have 15%. The thermal displacement ellipsoids shown in Fig. 2 ▸(*b*) indicate that this model in fact describes a continuous tetrahedral density, as there is considerable overlap of 8*c* and 32*f* densities. However, there are several signs that this model is insufficient. The refined occupancies in this model give an overall stoichiometry of Cu_1.72_Se. This is considerably lower than expected, as two crystals from the same synthesis batch have been found to have the composition Cu_1.95_Se (Eikeland *et al.*, 2017 ▸; Roth & Iversen, 2019 ▸). The residual density also shows missing density further along 〈111〉 from the 8*c* sites. There is also significant missing density between the 32*f* sites. The residual density is shown in Fig. S1 and also indicates that there is overlap of density between different Cu tetrahedra, which might indicate the diffusion pathway of Cu, further supporting that there is significant undescribed density in this model. The refinement *R* values (*R*_1_ = 4.15%, *wR*_2_ = 7.62%) also indicate that the model might be insufficient.

To improve the model description of the density, two more Cu sites are introduced, giving a four-site model. One more 32*f* site is used to describe the density further along the arms of the tetrahedron and one 48*g* site is used to describe the density between the arms of the tetrahedron. Again the occupancies are refined freely and anisotropic vibrational parameters are used. The position of the new 32*f* site is strongly correlated to the thermal parameter and occupancy, and the position was therefore fixed. This gives a total of 15 refined parameters. The resulting model is shown in Figs. 2 ▸(*c*) and 2 ▸(*d*), and all parameters are listed in the supporting information. As seen from Fig. 2 ▸(*c*), the occupancies are now distributed more over the different sites. The thermal ellipsoid of the new 32*f* site is highly elongated and describes the long-tailed behavior of the tetrahedron-shaped density, as observed in Fig. 2 ▸(*d*). The thermal ellipsoid of the new 48*g* site refines to an oblate shape, describing the density between two arms of the tetrahedron. Using this model the total stoichiometry refines to Cu_1.87_Se which is closer to the expected value, but still lower than expected. The refinement *R*-values have significantly improved to *R*_1_ = 2.34% and *wR*_2_ = 4.05%. The residual density has also improved significantly, as shown in the supporting information.

To test if further improvement of the model can be obtained, an extended four-site model is tested, in which anharmonic Gram–Charlier parameters are included on Se and the two most populated Cu sites. This gives a total of 35 parameters, which comes at the risk of overfitting the data, which has 115 unique observed reflections. The parameters obtained are listed in the supporting information. The refinement again improves the *R*-values to *R*_1_ = 1.23% and *wR*_2_ = 1.18%. The refined stoichiometry of this model is Cu_1.865_Se, which is almost identical to the four-site model. The residual density, shown in the supporting information, improves somewhat with this model, and is more featureless than for the four-site model. However, with the large amount of model parameters, it is possible that these improvements are partly a result of overfitting. An overview of the refinements can be seen in Table 1 ▸ and more details are given in the supporting information.

All these models are very simplified approximations to the real electron density, as they all approximate the continuous density by a number of separate sites. The purpose of these is to help show that the Cu density is continuous and more complex than can be described by normal models where discrete sites are used. The second purpose is to obtain the phases of observed reflections as well as the intensity of the 000 reflection. This makes it possible to carry out an accurate Fourier inversion to obtain the continuous electron density.

The average unit-cell electron density is then obtained by Fourier inversion of the measured amplitudes of the structure factors together with the refined phases. Because the structure is centro-symmetric, the phases only have two possibilities, ±1, which makes them more reliable than non-centrosymmetric crystals, where the phase can assume any value. All the refinement models used provide identical phases: all reflections except the 200 reflection are +1. The model also provides the 000 reflection, which cannot be measured, and the values for the different models are listed in Table 1 ▸. This reflection contributes with a constant background density, so a too-low refined stoichiometry will down-shift the resulting density, but does not change features present. Here the phases and 000 reflection from the four-site model are used.

**Figure 3:**
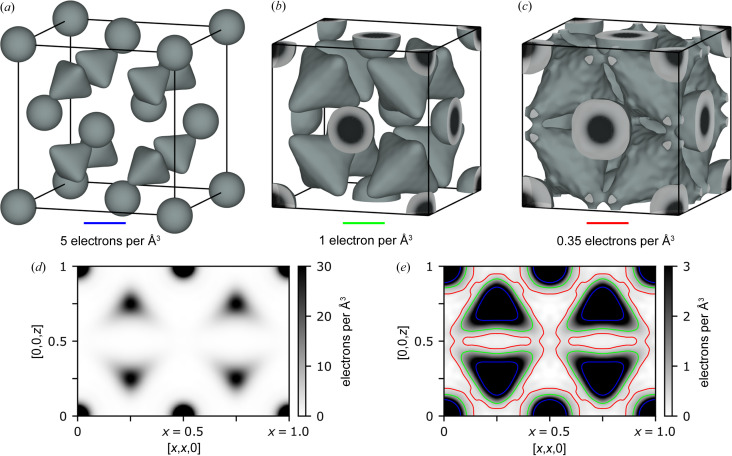
Observed electron density distribution in α-Cu_2−*x*_Se at 401 K obtained by Fourier inversion of observed amplitudes and model phases. (*a*), (*b*) and (*c*) show isosurfaces for 5, 1 and 0.35 electrons per Å^3^, respectively. (*d*) and (*e*) show the density in the [*x*,*x*,*z*] plane with different color ranges, and in (*e*) three contours are shown for the isosurfaces used. The effect of different models on the density is very small and is discussed in the supporting information.

Figs. 3 ▸(*a*) and 3 ▸(*b*) shows isosurfaces of the observed electron density for 5 and 1 electrons per Å^3^, respectively. It is observed that the Se density is largely spherical whereas the Cu density forms a tetrahedron-like shape with arms pointing towards the octahedral site. This is in agreement with previous studies (Dalgaard *et al.*, 2018 ▸; Oliveria, 1988 ▸; Yamamoto & Kashida, 1991 ▸), but with density further along the arms of the tetrahedron due to the higher resolution of data. A cut through the electron density in the 110 plane is shown in Fig. 3 ▸(*d*) for a large range in color scale, again showing the ‘arms’ of the Cu tetrahedra.

To further investigate the weak details of the density and to look for the possible diffusion pathway of Cu, a more narrow color range is shown in Fig. 3 ▸(*e*). The red contour line indicates 0.35 electrons per Å^3^ and shows a continuous channel of density between the Cu tetrahedra. This could suggest that the diffusion pathway goes between the two nearest Cu tetrahedra, starting from one tetrahedral 8*c* site and going along the 〈111〉 direction but avoids the octahedral site (which has a minimum density) by going along the 〈100〉 direction in the vicinity of the octahedral site and then along one of the 〈111〉 directions to another tetrahedral 8*c* site. This diffusion pathway is in agreement with that predicted theoretically by Danilkin *et al.* (2012 ▸). This possible channel is also visualized by the 0.35 electrons per Å^3^ isosurface shown in Fig. 3 ▸(*c*).

From Fig. 3 ▸ it is clear that most of the time Cu vibrates within a tetrahedron-like region around the 8*c* site and not in the channel between neighboring tetrahedra where the density is very low. Right at the 8*c* site the density is 32 electrons per Å^3^, while at the lowest point in the channel it is 0.4 electrons per Å^3^. The time spent between jumps from one tetrahedron to the next is therefore quite long. This is in qualitative agreement with the study by Voneshen *et al.* (2017 ▸) which found a long residence time of between 10 and 20 ps between jumps

Note that the low density of 0.4 electrons per Å^3^ at the minimum along the channel could be an artifact as a result of systematic errors or noise in the data, as these features are very weak. In Fig. 3 ▸(*c*) it is also observed that the 0.35 electrons per Å^3^ isosurface is not smooth, indicating that noise is present at this level. In the supporting information an even more narrow color range for the 110 plane is also shown, clearly indicating that the possible channel is not much stronger than the noise in the data.

In the field of multipole refinement of similarly high-quality single-crystal data, very weak features of 0.05 electrons per Å^3^ are used in the interpretation of data. However, in such cases they are also modeled using physically reasonable functions and are often stronger compared with the noise. See for example the analysis of weak van der Waals bonding in TiS_2_ measured at the same beamline (Kasai *et al.*, 2018 ▸), or the benchmarking study of this detector which uses the same setup, measurement types and data reduction procedures (Krause *et al.*, 2020 ▸).

To obtain a better indication of the uncertainties of the channels, the supporting information contains further analysis using the maximum entropy method (MEM) (Sakata & Sato, 1990 ▸; Iversen *et al.*, 1995 ▸; Smaalen *et al.*, 2003 ▸). The MEM analysis strongly indicates that there is a large uncertainty on the channels, corroborating that jumps between Cu sites are rare and that Cu does not move in the same manner as liquid-like ions through the structure. The presence of thermal diffuse scattering (TDS) might also make the observed electron density have sharper features than in reality. The ratio of TDS to Bragg intensity increases with increasing scattering vector, which can be problematic if not separated from the Bragg peaks during integration. If the integrated Bragg peaks include a TDS component which increases with *q*, it will give the apparent effect of lowering the overall thermal vibration. To avoid this most effectively, the experimental dataset was measured using fine-slicing (0.2°) to better allow the peak profile fitting in *SAINT* (Bruker, 2013 ▸) to separate Bragg intensity from TDS.

## 4. Correlated movements

From the measured total scattering (see Fig. 1 ▸), the diffuse scattering is isolated and Fourier transformed to obtain the 3D-ΔPDF. 3D-ΔPDF is the autocorrelation of the deviations in electron density from the average crystal structure:

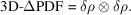

Here 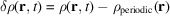 is the difference between the total electron density of the crystal and the periodic average electron density. 3D-ΔPDF provides a direct view of the local deviations from the periodic average structure (Weber & Simonov, 2012 ▸; Schaub *et al.*, 2007 ▸; Kobas *et al.*, 2005 ▸). Positive/negative features show vectors which separate atoms more/less often than in the average crystal structure. Different types and signs of features can, in simple cases, be directly related to the types of local correlations (Weber & Simonov, 2012 ▸). However, it is also possible to misinterpret features in the 3D-ΔPDF, and physical modeling should ideally be used to support the interpretation of features to avoid errors. 3D-ΔPDF has been used to understand the local order in several disordered crystals (Krogstad *et al.*, 2020 ▸; Roth & Iversen, 2019 ▸; Sangiorgio *et al.*, 2018 ▸; Zhang *et al.*, 2021 ▸; Roth *et al.*, 2020 ▸; 2021 ▸), and has also been extended to analyze local magnetic order in frustrated magnets (Roth *et al.*, 2019 ▸, 2018 ▸).

**Figure 4:**
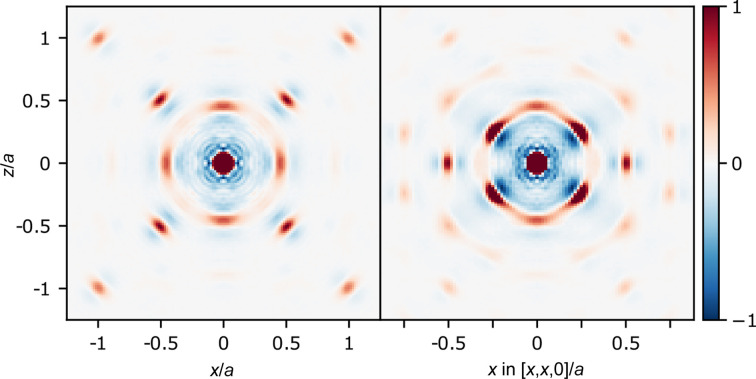
3D-ΔPDF for α-Cu_2−*x*_Se at 401 K in the 001 plane (left) and 110 plane (right).

Two planes of the 3D-ΔPDF for α-Cu_2−*x*_Se at 401 K are shown in Fig. 4 ▸. Before going into a more detailed analysis, a few general observations can be made. As usual there is a positive peak at the center with a negative feature around it, showing that each atom is not displaced from itself and there are no two atoms separated by very short distances, such as having two Cu ions inside the same tetrahedron at the same time. The other features have differing shapes, but they are overall comprised of some positive peak with negative regions at closer and longer distances. Furthermore, some of the positive peaks have long tails going in different directions, but without much change in the distance to the center. This shows that there are correlations of large movements, and that in general the distances between atoms do not change much, although the angles do (clearly observed by the arch-shaped positive regions in the 110 plane).

The signal from correlated movements contributes with a large factor to the 3D-ΔPDF. Because of the non-stoichiometry it would also be expected that there would be contributions from the vacancy distribution (substitution correlations) and from local relaxations around vacancies (size-effect correlations). There are no clear features of substitutional disorder directly visible in the 3D-ΔPDF. Substitutional correlations will give monopolar contributions to the 3D-ΔPDF, but they are often weaker than other contributions and might therefore not be directly visible. One possible way to analyze this is to integrate the features in the 3D-ΔPDF as was done in a recent study of a defective half-Heusler alloy (Roth *et al.*, 2021 ▸). However, this is not possible in this case as there is a high degree of overlap between peaks in the 3D-ΔPDF as a result of the large movements. Attempts to do so produces unphysical results, which are in strong contradiction to the average crystal structure, as discussed in the supporting information. Another approach would be to measure energy-resolved diffuse neutron scattering, as the diffuse scattering resulting from short-range vacancy order should occur at lower energies than the strong diffuse scattering from correlated movements. However, as most of the diffuse scattering has been found to be quasi-elastic, it might be difficult to obtain a large enough reciprocal space coverage with good enough energy-resolution using current instruments.

The typical size-effect signatures in a 3D-ΔPDF are dipolar features (Weber & Simonov, 2012 ▸; Roth *et al.*, 2021 ▸). The feature found for the Cu–Se vector (1/4,1/4,1/4), observed in the right part of Fig. 4 ▸, has what appears to be a dipolar component as it has a large positive contribution away from the origin and a strong negative contribution towards the origin. This has the same signature as a typical sign of local relaxations corresponding to Se moving closer to a vacant Cu site than to an occupied one. The scattering itself also shows a typical signature of size-effect, which is diffuse scattering being stronger on one side of a Bragg peak than the other (Welberry, 2004 ▸). This is most evident for the [*hh*0]-type reflections, as observed in both panels of Fig. 1 ▸ (diffuse features are stronger towards the center of reciprocal space for these reflections). However, with such an anharmonic system, those features might also originate from complex correlated anharmonic movements. When dealing with anharmonic systems, a clear distinction between size-effect and displacement disorder is not possible.

To make sure the interpretation of the 3D-ΔPDF is correct, some type of modeling should ideally be used to support claims made from direct inspection of the 3D-ΔPDF, as it can be misinterpreted, especially in complex systems with several contributions to the diffuse scattering. This can either be in the form of a simulated scattering pattern and 3D-ΔPDF from a physically sensible model in agreement with the average crystal structure, or from refinement of correlation parameters to the data. However due to the large degree of anharmonicity in the movement of Cu, as evident from the tetrahedral density, the refinement program *YELL* (Simonov *et al.*, 2014 ▸) cannot be used, as it currently only supports harmonic vibrations.

**Figure 5:**
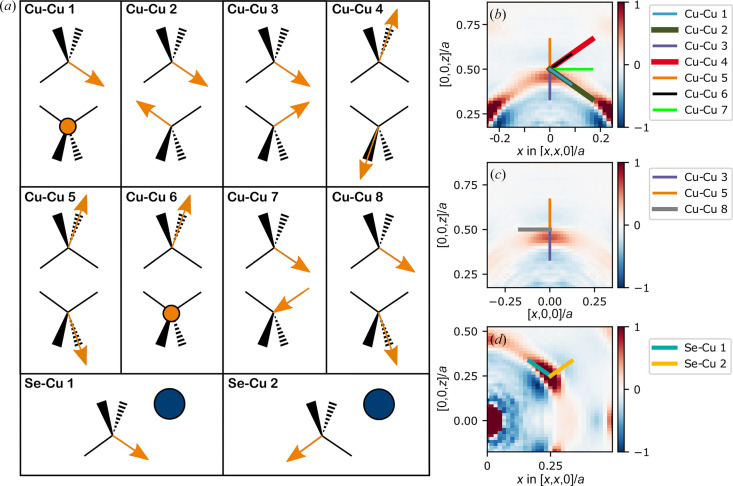
Simplified correlated movements. (*a*) Some different possible simplified movements. Each Cu ion is located on a tetrahedron. An orange arrow represents a Cu ion moving from the center towards a corner of the tetrahedron. An orange circle is a stationary Cu ion. A blue circle is a stationary Se ion. Wedged and dashed lines show movement in and out of the plane, respectively. (*b*)–(*d*) Traces of the different movements in the 3D-ΔPDF for 0 < *t* < 1/3.

Instead a different approach is needed to obtain some qualitative information about the correlated movements. A simplified model for the Cu movements is used, where each Cu is either positioned at the tetrahedral 8*c* site, or moving from the 8*c* site along one of the tetrahedral arms in the directions of an octahedral 4*b* site. This movement is parametrized using 0 ≤ *t* ≤ 1, which is the extent of the movement; *t* = 0 corresponds to the atom at the tetrahedral site and *t* = 1 at the octahedral site. A number of possible such correlated movements of neighboring Cu ions are shown schematically in Fig. 5 ▸(*a*). Each panel in the figure shows two neighboring Cu ions in the 110 plane. An orange arrow represents a Cu ion moving from the center towards a corner of the tetrahedron. An orange circle is a stationary Cu ion. Wedged and dashed lines show movement in and out of the plane, respectively. The figure also shows two possible types of movements of a Cu ion in relation to the nearest Se ion, illustrated as a blue circle.

If some of these movement types are present more frequently than would be expected for uncorrelated movements, the trace of the interatomic vector for such a movement would show a positive line in the 3D-ΔPDF. The parametrized trace of the interatomic vectors for these movement types are given in Table 2 ▸. As seen from the table, most of these movements, except ‘Cu–Cu 8’ produce traces in the 110 plane. The ‘Cu–Cu 8’ movement will produce a trace in the 001 plane, together with the traces of ‘Cu–Cu 3’ and ‘Cu–Cu 5’, which are in both planes.

Figs. 5 ▸(*b*), 5 ▸(*c*) and 5 ▸(*d*) show the zoomed-in regions of the 3D-ΔPDF for nearest Cu–Cu and Cu–Se interactions. The traces for the different possible movement types are shown on top with colored lines. The traces are only shown for 0 ≤ *t* ≤ 1/3, corresponding to Cu movements going 1/3 of the way from the tetrahedral site to the octahedral site, owing to the Cu ions spending most of the time in this region, evident from Fig. 3 ▸.

The movements of types ‘Cu–Cu 4’, ‘Cu–Cu 5’ and ‘Cu–Cu 6’ all go through negative regions of the 3D-ΔPDF, suggesting that they do not occur very often in the real structure. These are the movements which make the interatomic distance longer. On the other hand, the movement types ‘Cu–Cu 1’ and ‘Cu–Cu 2’ follow the positive lines in the 3D-ΔPDF somewhat, suggesting that movements of these types could be frequent in the real structure. ‘Cu–Cu 1’ and ‘Cu–Cu 2’ are quite similar types of movements, where the difference is that one of the ions in ‘Cu–Cu 1’ is stationary while both Cu ions move together in ‘Cu–Cu 2’. Linear combinations of these, corresponding to one of the ions moving to a lesser degree than the other, would also give traces along the positive region in the 3D-ΔPDF. The movement of type ‘Cu–Cu 3’ allows two Cu ions to become closer. At a short distance it passes through the positive part of the peak, whereas at longer distances it moves into a negative region, suggesting that slight contraction of the Cu–Cu distances might occur, but only to a certain extent. Movements of type ‘Cu–Cu 7’ and ‘Cu–Cu 8’ give slightly longer distances between Cu ions. The traces of these movements go though regions where the 3D-ΔPDF is approximately zero. This suggest that these types of movements could occur to some degree, on a similar scale that which would occur if the movements were uncorrelated.

Each Cu has four Se neighbors. No matter which of the arms in the tetrahedron the Cu moves along, a movement of type ‘Se–Cu 1’ will take place with regards to three of the arms, while a movement of the ‘Se–Cu 2’ type will do so with reagrds to one. ‘Se–Cu 2’, with a longer distance between Se and Cu, has a trace in the negative region, whereas ‘Se–Cu 1’, which almost keeps the same distance between the two, has a trace in the positive region. This suggests that when the Cu ion moves along one of the arms of the tetrahedron, the Se which it moves away from will follow the Cu ion to roughly maintain the distance. The strong negative feature towards the origin shows a strong preference for Cu and Se to not move any closer than their ideal bond length.

The shortest Cu–Cu and Cu–Se vectors in the structure are unique, which allows for some considerations of the typical types of movements in this system. However, further analysis such as the Se–Se correlations are not possible using this simplified approach as the nearest Se–Se vector is identical to the next-nearest Cu–Cu vector. The analysis of systems with such strong correlated anharmonicity such as this case is currently limited, as there are currently no established methods of modeling such behavior using 3D-ΔPDF. An alternative route is to simulate the system using molecular dynamics (MD) to extract correlations, or possibly simulate 3D-ΔPDF data from MD to compare with the experiment.

## 5. Conclusions

Through a combination of analysis of high-quality Bragg diffraction data and diffuse scattering data, the structure and correlations of superionic conductor α-Cu_2−*x*_Se have been investigated. The system is an example of a material where the crystal structure is more than just a list of atomic positions. A density-based description is needed to describe the large movements and channels in the structure. The Se ions form an f.c.c. lattice with the Cu ions thermally distributed around the tetrahedral sites, to form a modified anti-fluorite structure. The Cu ions mainly occupy a volume forming a tetrahedron with arms pointing towards the empty octahedral sites, showing extreme anharmonicity in the Cu vibrations. From analysis of the very weak features in the observed density, the diffusion of Cu ions is suggested to take place through channels in the structure, moving from the tetrahedral site towards the octahedral site along the 〈111〉 direction, but avoiding the octahedral site by moving around it in its vicinity, which is in qualitative agreement with the predicted pathway from theoretical considerations by Danilkin *et al.* (2012 ▸). However, the density in the channel is very low compared with the region around the tetrahedral site, showing that jumps between sites are infrequent compared with the time each Cu ion spends in the tetrahedron-like region. This is in agreement with the results by Voneshen *et al.* (2017 ▸) who found a long residence time between diffusion-contributing jumps of Cu ions. This further supports their interpretation, which is that α-Cu_2−*x*_Se has an extreme amount of anharmonicity which might be the origin of the low thermal conductivity, instead of a liquid-like Cu substructure. Although there is diffusion of Cu ions in the structure, making it a superionic conductor, the jumps are infrequent and probably not the origin of the low thermal conductivity.

From the 3D-ΔPDF analysis of the diffuse scattering, we found that there are strong correlations between the large movements of Cu ions in the structure, as well as strong correlations between Cu and Se. In general, these are movements which change the angles between atoms considerably, but keep distances almost unchanged. Due to the large anharmonic motions, current diffuse scattering modeling methods cannot be applied, limiting what can be stated with certainty about the correlations in the structure.

## Supplementary Material

Additional supporting data and analysis. DOI: 10.1107/S2052252523001318/cx5005sup1.pdf

## Figures and Tables

**Table 1:** Measurement details, data quality indicators and model refinement indicators List of all measured intensities, structure factors and number of observations as well as model parameters can be found in the supporting information.

Measurement and data			Refinement models			
Space group	*Fm* 3 *m*		Model	Two-site	Four-site	Ext. Four-site
Temperature	401 K		Stoichiometry	Cu_1.717_Se	Cu_1.87_Se	Cu_1.865_Se
*a* (Å)	5.8686 (8)		*F*(000)	334	353	352
λ (Å)	0.248		*N*_parameters_	7	15	35
*d*_min_ (Å)	0.403		*R*_1_	0.0415	0.0234	0.0123
*N*_Tot,obs_ (I > 3σ)	11303		*wR*_2_	0.0762	0.0405	0.0118
*N*_Uniq,obs_	115					
*R*_int_	0.0306					

**Table 2:** Traces of different simplified movements in α-Cu_2−*x*_Se The parameter 0 < *t* < 1 is the extent of the movement, where *t* = 0 corresponds to the atom at the tetrahedral site and *t* = 1 the atom at the octahedral site. See Fig. 5 ▸ for a definition of the different movement types.

Movement type	Trace in 3D-ΔPDF
Cu–Cu 1	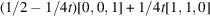
Cu–Cu 2	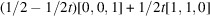
Cu–Cu 3	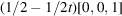
Cu–Cu 4	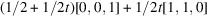
Cu–Cu 5	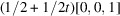
Cu–Cu 6	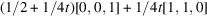
Cu–Cu 7	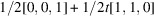
Cu–Cu 8	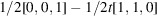
Se–Cu 1	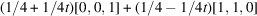
Se–Cu 2