# Supplementary material for: Dynamic correlations and possible diffusion pathway in the superionic conductor Cu2−x Se
Source: IUCrJ. 2023 Feb 17;10(Pt 2):199–209. doi: 10.1107/S2052252523001318 (PMC9980382; doi:10.1107/S2052252523001318)
Supplement: Supplementary file 1 [file m-10-00199-sup1.pdf]

IUCrJ

Volume 10 (2023)

Supporting information for article:

Dynamic correlations and possible diffusion pathway in superionic conductor, Cu2-xSe

Nikolaj Roth and Bo Brummerstedt Iversen

Residual density plots

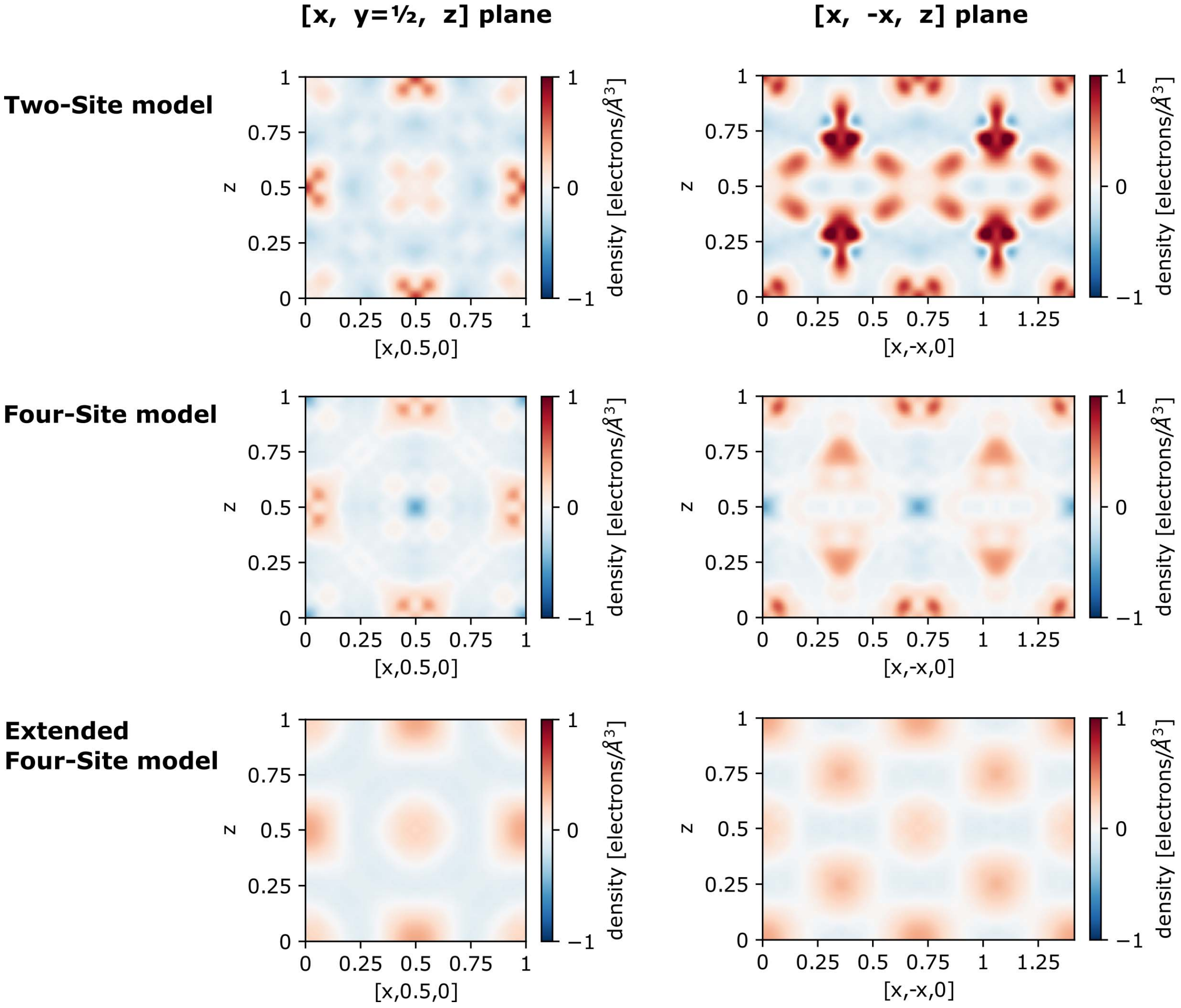

Figure S1. Residual density (obs-calc) for the different refinement models.

Model bias on the observed density

The model is used to phase the observed amplitudes, as well as to give the 000-reflection amplitude. Therefore the model can effect the Fourier inversion of the observed structure factors. Figure S2 shows the Fourier inversion densities from the observed structure factors phases by the different models.

All models give the same phases to all reflections (all are +1 except for the 002 reflection which is -1). This means that the only difference between the models is the F(000) amplitude, which contributes with a constant average density. In general all models result in very similar observed densities, but there are weak differences on the level ~0.1 electrons/Å^3. For the two-site model, negative densities are observed between atoms. This is unphysical and shows that the refined stoichiometry is too low (as this gives a too low F(000)). As the Four-site model and extended four-site model give almost identical F(000), the densities obtained are also almost identical.

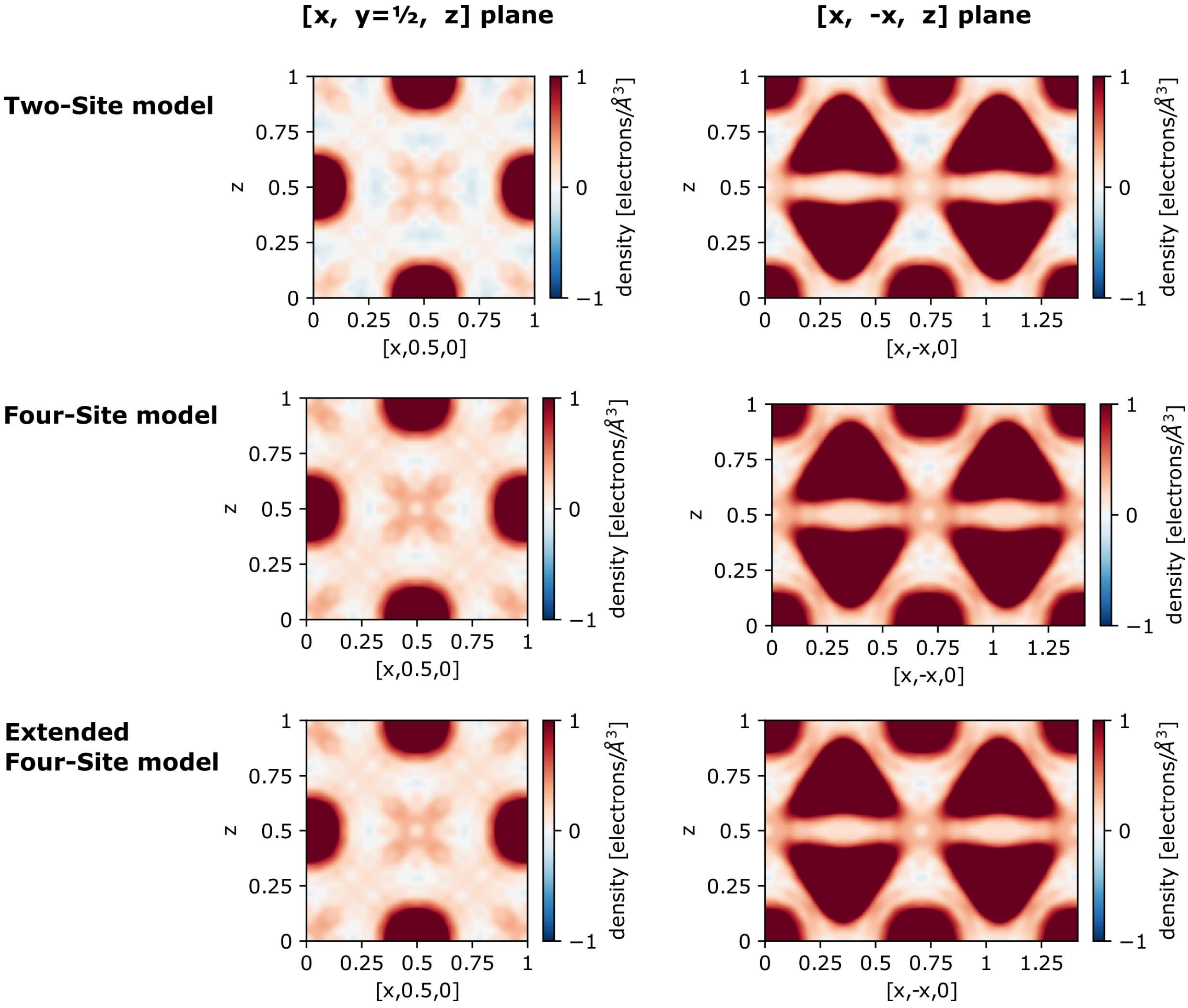

Figure S2. Fourier inversion of **observed** structure factors for the different models.

Effects of Punch type and size

The method used to separate Bragg peaks from the diffuse scattering has an effect on the resulting 3D-ΔPDF. If too little intensity is removed from the Bragg positions, positive peaks will be seen for all interatomic vectors in the 3D-ΔPDF, as this is essentially the same as partially adding the Patterson function to the 3D-ΔPDF. This will also result in a large positive integral over the whole 3D-ΔPDF. If too much scattering is removed around the Bragg positions, it will typically result in the addition of spurious negative peaks to the 3D-ΔPDF, which can be hard to distinguish from negative features in the 3D-ΔPDF, which are real. In cases where the diffuse scattering peaks at the Bragg position, removal of too much diffuse scattering will also result in a too fast decay of the 3D-ΔPDF, as the high-frequency components of a diffuse peak are removed.

Figure S3 shows the total scattering in the HK0 and HHL planes, as well as the scattering obtained after punch and fill and the resulting 3D-ΔPDFs. The top row shows the total scattering, with strong and sharp Bragg peaks at symmetry-allowed integer H, K and L positions, as well as spread-out diffuse scattering. The diffuse scattering peaks at the Bragg positions, and is particularly strong around the [2,2,0] , [4,0,0] and [4,4,0] reflections. The second row shows the result of using a small punch on all allowed Bragg positions of the space-group. This removes most of the Bragg scattering, but leaves very strong peaks at positions such as [1,1,1], suggesting that the small punch size was not sufficient to catch all Bragg peaks. The third row shows the result of using a larger punch size. Although this removes all Bragg peaks, it is also clear that it removes too much of the diffuse scattering. E.g. there is a clear problem with the [2,2,0] peaks, where too much has been removed at the Bragg position, leaving a small "pinch" after filling. The 3D-ΔPDF now shows negative peaks at positions which were not negative before. As too much scattering around Bragg peaks was removed, these negative peaks are probably not real, but a consequence of having "holes" in the scattering at the Bragg positions. The fourth row shows the result of using a frame-by-frame removal of sharp and intense features as described in the experimental section. This removes much of the Bragg scattering, but is not effective at removing weak peaks due to the necessity of a threshold value to not remove noise. The 3D-ΔPDF in this case now has positive peaks at many interatomic vectors, further showing that some Bragg intensity is still left. The bottom row shows the combination of using the frame-by-frame peak removal together with the small punch size, to catch the remaining weak peaks left after the frame-by-frame peak removal. This produces scattering data where the Bragg peaks have been removed, but without clear "pinches" or holes at the Bragg positions. It furthermore produces more clean 3D-ΔPDF maps. The top right part of the figure shows line-cuts along the [4,4,L] line of the scattering, showing the effect of the different methods on selected Bragg peaks. From the line cuts the same essential points can be seen, although some of the effects are less clear when only looking along one dimension.

One should be cautious when interpreting parts of the 3D-ΔPDF which depend of the punch size and method. In this case the features interpreted in the paper are those which do not change with the punch size and method.

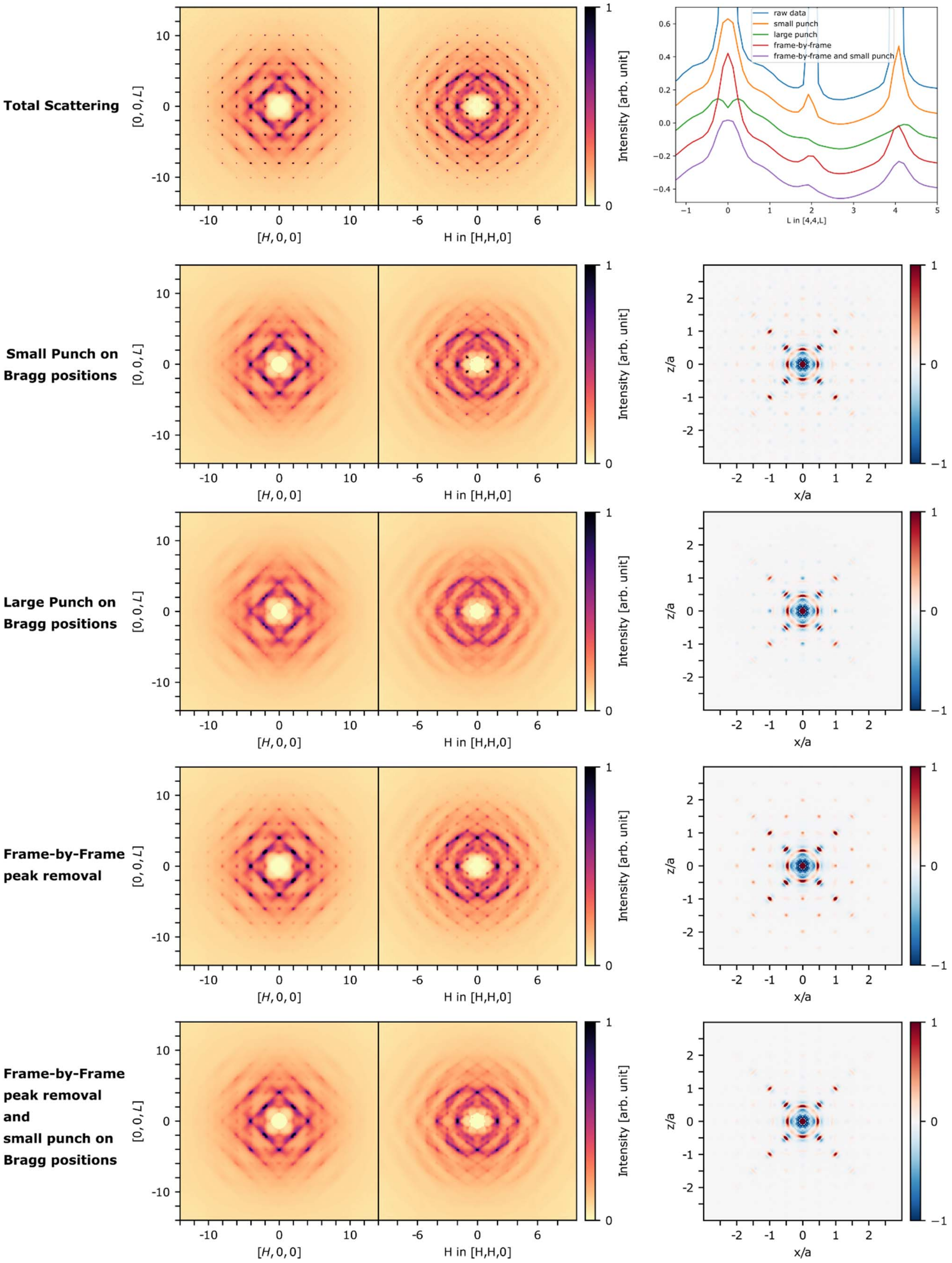

Figure S3. Different punching methods and their effect on the 3D-ΔPDF

Problem with overlap of 3D-ΔPDF peaks in determining integral amplitudes

Due to a large overlap of features in the 3D-ΔPDF, it was not possible to isolate the substitutional contribution by integration of features. The top row of Figure S4 shows an attempt at making integration basins around features. Here, all points nearest to an interatomic vector of the ideal antiflourite structure is assigned to the integration basin of that feature. Black lines are used to illustrate the boundary of features.

A clear illustration of the problem of overlap in seen for the features at (0,0,½) and (1/4,1/4,1/4), visible in the HHL plane (top right in Figure S4). Both of these features have negative contributions towards the center of space. The results of integration of these basins is shown in the bottom part of the figure. The feature at (0,0,½) is positive and the feature at (1/4,1/4,1/4) is negative. The (1/4,1/4,1/4) vector is the vector between Cu and Se. If real, the negative integral of the (1/4,1/4,1/4) feature would suggest that this vector separates fewer atoms than in the average structure. This could only happen if there was missing Se in the structure, and Cu and Se vacancies tended to avoid each other. However, there is no indication that there should be any missing Se, and the negative integral of this peak is more likely a result overlap of features, such that the integration basin of (1/4,1/4,1/4) has absorbed much of the negative lobe of the (0,0,½) feature.

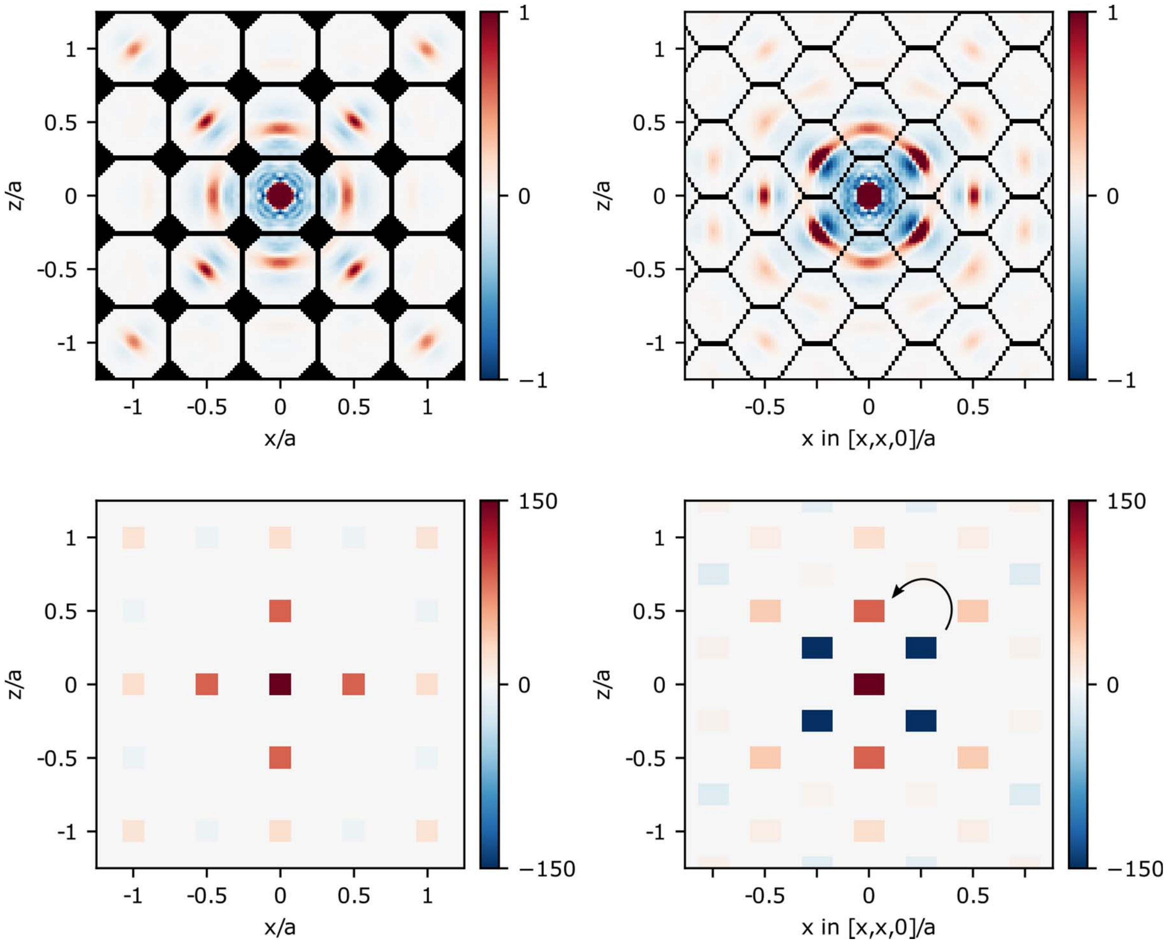

Figure S4. Attempt at integration of features in the 3D-ΔPDF

Maximum Entropy Method

Fourier inversion shows very weak channels in the structure between Cu sites. To get a better estimate on the uncertainty on these channels, Maximum Entropy Method (MEM) is used. MEM gives the electron density of the unit cell based on the phased structure factors and their errors. It is not dependent on a structural model other than through the phasing of reflections, which is this case is robust for all models. MEM gives the most unbiased electron density consistent with the structure factors (Sakata & Sato, 1990). MEM calculations were performed in the BayMEM software (Smaalen et al., 2003) with the Sakato–Sato algorithm (Sakato & Sato, 1990). A grid of 128×128×128 voxels was used together with a flat prior density. Convergence was obtained at $\chi^2 = 1$.

Figure S5 shows the electron density in the xxz plane obtained from Fourier Inversion (FI) and MEM. While the FI density shows very weak channels between Cu sites, the MEM density does not. FI will usually be biased if not all non-zero reflections are included. But in this case the scattering measurement extended much further than any observable reflections as discussed in the main text. This suggests that the channels seen in the FI has a large uncertainty, as the $\chi^2 = 1$ MEM does not contain them. MEM converged to a lower stopping value of χ^2 will eventually produce the same features as the FI density. Using $\chi^2 = 1$ as a stopping value assumes that the estimated standard deviations (ESDs) are correct. In reality this is not always the case, and if ESDs are overestimated, a $\chi^2 = 1$ MEM density will produce a more smeared-out density compared to the real density.

The MEM density strongly indicates that there is a large uncertainty on the channels observed in the FI density, further corroborating that jumps between Cu sites are rare and that Cu does not move as liquid-like ions through the structure.

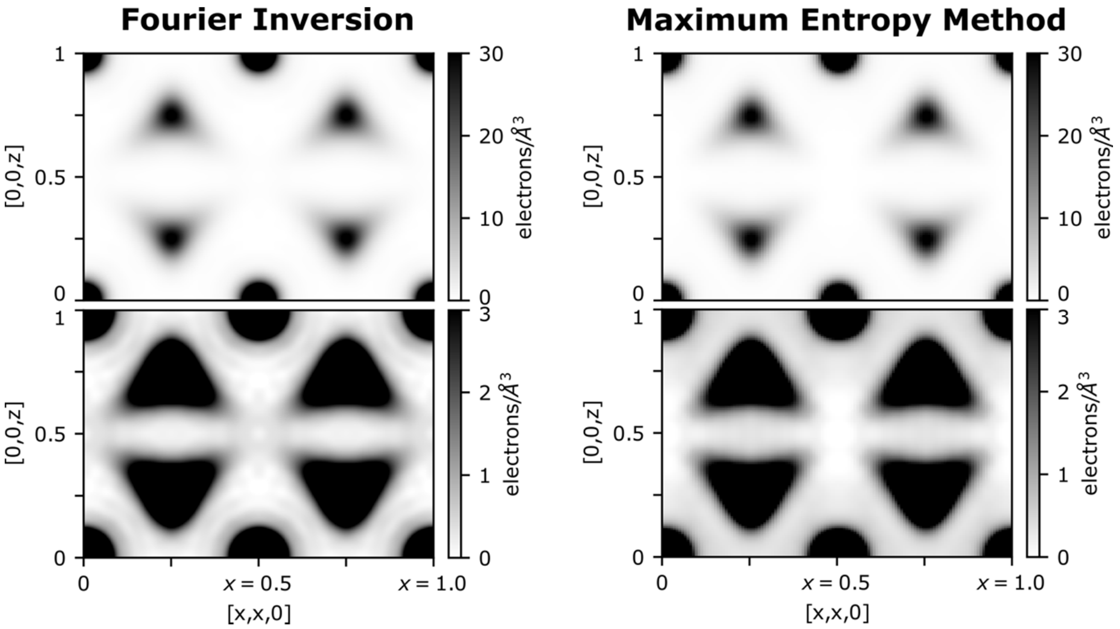

Figure S5. Electron density from Fourier inversion and Maximum Entropy Method.

Refinement Models

Table S1. Refinement parameters and indicators for the different models

Two-Site Model

Stoichiometry	$Cu_{1.717}Se$
F(000)	334
$N_{parameters}$	7
R_1	0.0415
wR_2	0.0762

Site	Type	x	y	z	Therm. Param. Type	Uiso/Ueqv	occ.
Se1	Se	0	0	0	Isotropic	0.03715(14)	1
Cu0	Cu	0.25	0.25	0.25	Isotropic	0.056(5)	0.26(11)
Cu1	Cu	0.304(9)	0.304(9)	0.304(9)	Anisotropic	0.083(10)	0.15(3)

Anisotropic Thermal Parameters

Site	Type	U11	U22	U33	U12	U13	U23
Cu1	Cu	0.083(18)	0.083(18)	0.083(18)	0.036(18)	0.036(18)	0.036(18)

Four-Site Model

Stoichiometry	$Cu_{1.87}Se$
F(000)	353
$N_{parameters}$	15
R_1	0.0234
wR_2	0.0405

Site	Type	x	y	z	Therm. Param. Type	Uiso/Ueqv	occ.
Se1	Se	0	0	0	Isotropic	0.03708(7)	1
Cu0	Cu	0.25	0.25	0.25	Isotropic	0.054(5)	0.16(7)
Cu1	Cu	0.326(19)	0.326(19)	0.326(19)	Anisotropic	0.075(13)	0.07(5)
Cu2	Cu	0.4	0.4	0.4	Anisotropic	0.09(2)	0.013(7)
Cub	Cu	0.25	0.296(6)	0.25	Anisotropic	0.071(11)	0.07(3)

Anisotropic Thermal Parameters

Site	Type	U11	U22	U33	U12	U13	U23
Cu1	Cu	0.08(2)	0.08(2)	0.08(2)	0.03(2)	0.03(2)	0.03(2)

Cu2	Cu	0.09(4)	0.09(4)	0.09(4)	0.05(4)	0.05(4)	0.05(4)
Cub	Cu	0.07(2)	0.073(9)	0.07(2)	0	0.04(2)	0

Extended Four-Site model

Stoichiometry	$Cu_{1.865}Se$
F(000)	352
$N_{parameters}$	35
R_1	0.0123
wR_2	0.0118

Site	Type	x	y	z	Therm. Param. Type	Uiso/Ueqv	occ.
Se1	Se	0	0	0	Anharmonic	0.03650(12)	1
Cu0	Cu	0.25	0.25	0.25	Anharmonic	0.057(2)	0.37(6)
Cu1	Cu	0.319(4)	0.319(4)	0.319(4)	Anharmonic	0.057(4)	0.11(2)
Cu2	Cu	0.400255	0.400255	0.400255	Anisotropic	0.066(12)	0.008(5)
Cub	Cu	0.25	0.35(4)	0.25	Anisotropic	0.15(5)	0.018(12)

Anisotropic Thermal Parameters

Site	Type	U11	U22	U33	U12	U13	U23
Se1	Se	0.0365(2)	0.0365(2)	0.0365(2)	0	0	0
Cu0	Cu	0.057(4)	0.057(4)	0.057(4)	0	0	0
Cu1	Cu	0.057(6)	0.057(6)	0.057(6)	0.019(6)	0.019(6)	0.019(6)
Cu2	Cu	0.07(2)	0.07(2)	0.07(2)	0.03(2)	0.03(2)	0.03(2)
Cub	Cu	0.15(9)	0.15(8)	0.15(9)	0	0.12(9)	0

Gram-Charlier Parameters (Only unique values)

Site	Type	D1111	D1122	F111111	F111122	F112233
Se1	Se	-0.0022(3)	-0.00020(13)	-0.00064(14)	-0.00015(2)	-0.000008(16)

Site	Type	C123	D1111	D1122
Cu0	Cu	-0.003(8)	0.008(3)	-0.005(2)

Site	Type	C111	C112	D1111	D1112	D1122	D1123
Cu1	Cu	0.07(3)	0.04(3)	-0.021(8)	-0.013(7)	-0.010(7)	-0.008(6)

Reflection Intensities, statistics and Structure factors

H	K	L	I (unscaled)	σ(I)	I/σ	F (scaled and phased)	σ(F)	$N_{measured}$
1	1	1	2983.21	70.0074	42.6	101.01000	1.5574	13
2	0	0	99.8845	2.35461	42.4	-18.48000	0.28538	33
2	2	0	9434.45	216.168	43.6	179.63001	2.7318	22
2	2	2	42.3715	1.00192	42.3	12.02800	0.18621	20
3	1	1	2237.21	50.6964	44.1	87.47000	1.322	30
3	3	1	560.635	12.7085	44.1	43.78400	0.66194	133
3	3	3	1189.73	27.5058	43.3	63.78500	0.97514	12
4	0	0	2670.15	65.3941	40.8	95.55900	1.5109	5
4	2	0	137.97	3.11804	44.2	21.71700	0.32791	137
4	2	2	1655.78	37.3554	44.3	75.24900	1.1345	44
4	4	0	1065.68	25.3788	42.0	60.36700	0.93882	18
4	4	2	101.05	2.27775	44.4	18.58600	0.28028	163
4	4	4	215.734	4.88206	44.2	27.15900	0.41011	47
5	1	1	533.987	11.9868	44.5	42.73100	0.64255	141
5	3	1	420.475	9.43319	44.6	37.91800	0.5698	296
5	3	3	100.243	2.26042	44.3	18.51200	0.27903	159
5	5	1	83.2027	1.88006	44.3	16.86500	0.25453	149
5	5	3	135.544	3.04581	44.5	21.52700	0.32411	163
5	5	5	16.092	0.37137	43.3	7.41540	0.11305	35
6	0	0	177.19	4.02436	44.0	24.61300	0.37229	43
6	2	0	385.962	8.66823	44.5	36.32800	0.54641	150
6	2	2	87.8967	1.98804	44.2	17.33500	0.2619	149
6	4	0	40.5414	0.92429	43.9	11.77200	0.1781	168
6	4	2	162.189	3.63551	44.6	23.54800	0.35402	300
6	4	4	31.2094	0.71456	43.7	10.32800	0.15648	148
6	6	0	77.4788	1.77095	43.7	16.27500	0.24714	57
6	6	2	14.7326	0.3356	43.9	7.09500	0.10839	106
6	6	4	17.1217	0.38918	44.0	7.64930	0.11599	85
6	6	6	5.38624	0.13556	39.7	4.29150	0.070379	29
7	1	1	120.5	2.71376	44.4	20.29700	0.30551	154
7	3	1	73.7513	1.65693	44.5	15.87800	0.23912	317

7	3	3	58.8922	1.33066	44.3	14.18900	0.21408	157
7	5	1	36.8345	0.82707	44.5	11.22000	0.16909	215
7	5	3	11.291	0.25456	44.4	6.21130	0.092712	195
7	5	5	12.9409	0.29515	43.8	6.65010	0.10185	75
7	7	1	5.66046	0.13056	43.4	4.39760	0.067002	108
7	7	3	8.85213	0.20296	43.6	5.49950	0.083019	98
7	7	5	1.48136	0.03855	38.4	2.24830	0.037824	57
7	7	7	1.04521	0.03838	27.2	1.89390	0.04077	19
8	0	0	68.9674	1.63084	42.3	15.35500	0.23777	41
8	2	0	32.026	0.73317	43.7	10.46300	0.15869	146
8	2	2	47.0583	1.06144	44.3	12.68300	0.19109	107
8	4	0	32.2304	0.72728	44.3	10.49600	0.15863	110
8	4	2	10.7813	0.24328	44.3	6.06940	0.090865	177
8	4	4	13.5928	0.3108	43.7	6.81510	0.10342	95
8	6	0	3.52381	0.08389	42.0	3.46770	0.052524	111
8	6	2	9.62821	0.21791	44.2	5.73670	0.08713	201
8	6	4	2.83678	0.06633	42.8	3.11490	0.049465	184
8	6	6	1.21053	0.03231	37.5	2.03300	0.032402	62
8	8	0	3.60206	0.09318	38.7	3.50730	0.056173	32
8	8	2	0.93873	0.02645	35.5	1.79170	0.033768	68
8	8	4	0.86452	0.02537	34.1	1.71390	0.034483	61
8	8	6	0.38774	0.01803	21.5	1.15410	0.031787	56
8	8	8	0.08904	0.0363	2.5	0.55426	0.12342	15
9	1	1	17.973	0.40845	44.0	7.83690	0.11894	99
9	3	1	11.8541	0.26749	44.3	6.36380	0.096508	175
9	3	3	7.62087	0.17424	43.7	5.10280	0.076483	104
9	5	1	5.05108	0.11481	44.0	4.15390	0.061451	227
9	5	3	4.29019	0.09815	43.7	3.82860	0.058827	211
9	5	5	0.95293	0.0279	34.2	1.80120	0.033691	57
9	7	1	1.92407	0.04538	42.4	2.56110	0.042073	132
9	7	3	0.62671	0.01793	35.0	1.46670	0.027539	125
9	7	5	0.76415	0.02037	37.5	1.61120	0.026646	125
9	7	7	0.12775	0.01586	8.1	0.66614	0.051726	55

9	9	1	0.25091	0.01639	15.3	0.92386	0.038127	61
9	9	3	0.38985	0.01648	23.7	1.15410	0.031787	67
9	9	5	0.08872	0.01493	5.9	0.55422	0.03132	62
9	9	7	0.07366	0.02073	3.6	0.48884	0.070074	48
10	0	0	5.57952	0.13879	40.2	4.36650	0.070088	30
10	2	0	6.74021	0.15474	43.6	4.79920	0.071833	90
10	2	2	3.79357	0.09019	42.1	3.59850	0.055892	96
10	4	0	2.51363	0.06149	40.9	2.92830	0.045662	110
10	4	2	3.20553	0.07383	43.4	3.31170	0.049023	221
10	4	4	0.97106	0.02699	36.0	1.82010	0.033544	68
10	6	0	1.54951	0.03879	39.9	2.30110	0.037588	71
10	6	2	0.67966	0.01881	36.1	1.52380	0.027122	129
10	6	4	0.72539	0.0194	37.4	1.57910	0.0268	130
10	6	6	0.22187	0.01595	13.9	0.86669	0.040373	65
10	8	0	0.16616	0.01511	11.0	0.76173	0.045499	61
10	8	2	0.40424	0.01386	29.2	1.16880	0.018724	132
10	8	4	0.17195	0.01112	15.5	0.76182	0.023689	121
10	8	6	0.05915	0.01179	5.0	0.45254	0.038023	112
10	10	0	0.09647	0.0314	3.1	0.58437	0.087922	26
10	10	2	0.03263	0.01911	1.7	0.31995	0.10683	40
11	1	1	1.96693	0.04897	40.2	2.59420	0.041941	49
11	3	1	1.38817	0.03348	41.5	2.17900	0.03208	128
11	3	3	0.97506	0.02761	35.3	1.82960	0.033474	61
11	5	1	0.67954	0.01869	36.4	1.52400	0.027122	130
11	5	3	0.42535	0.01421	29.9	1.21180	0.0186	133
11	5	5	0.2866	0.01549	18.5	0.99516	0.035758	62
11	7	1	0.23162	0.01154	20.1	0.88619	0.021224	129
11	7	3	0.20233	0.01096	18.5	0.82639	0.02227	127
11	7	5	0.05367	0.01231	4.4	0.41306	0.041561	105
11	9	1	0.09019	0.01267	7.1	0.55430	0.03132	92
11	9	3	0.04967	0.01483	3.3	0.41304	0.041561	80
12	0	0	0.731	0.03805	19.2	1.57910	0.046086	13
12	2	0	0.47768	0.01887	25.3	1.28030	0.029611	61

12	2	2	0.55108	0.01961	28.1	1.37060	0.028461	65
12	4	0	0.36158	0.01708	21.2	1.10880	0.032761	61
12	4	2	0.24803	0.01173	21.1	0.92392	0.020678	132
12	4	4	0.20115	0.01444	13.9	0.82641	0.02227	66
12	6	0	0.13561	0.01536	8.8	0.69135	0.049911	63
12	6	2	0.181	0.01071	16.9	0.78396	0.023165	131
12	6	4	0.05714	0.01191	4.8	0.45251	0.038023	104
12	8	0	0.086	0.0187	4.6	0.55429	0.061898	43
12	8	2	0.01249	0.0142	0.9	0.18473	0.092491	79
13	1	1	0.16259	0.01532	10.6	0.73914	0.046824	58
13	3	1	0.11836	0.01147	10.3	0.64009	0.027453	123
13	3	3	0.08747	0.01582	5.5	0.55431	0.061898	55
13	5	1	0.07313	0.01105	6.6	0.48884	0.035292	119
13	5	3	0.06412	0.01271	5.0	0.45256	0.038023	101
14	0	0	0.09762	0.03216	3.0	0.58424	0.087922	14
14	2	0	0.05069	0.01881	2.7	0.41315	0.082813	50
14	2	2	0.02812	0.01847	1.5	0.31999	0.10683	44
14	4	0	0.01152	0.01902	0.6	0.18474	0.18495	46
